# Fetal growth restriction adversely impacts trajectory of hippocampal neurodevelopment and function

**DOI:** 10.1111/bpa.13330

**Published:** 2025-01-08

**Authors:** Ingrid Dudink, Amy E. Sutherland, Margie Castillo‐Melendez, Elham Ahmadzadeh, Tegan A. White, Atul Malhotra, Harold A. Coleman, Helena C. Parkington, Justin M. Dean, Yen Pham, Tamara Yawno, Tara Sepehrizadeh, Graham Jenkin, Emily J. Camm, Beth J. Allison, Suzanne L. Miller

**Affiliations:** ^1^ The Ritchie Centre Hudson Institute of Medical Research, Translational Research Facility Clayton VIC Australia; ^2^ Department of Obstetrics and Gynaecology Monash University Clayton VIC Australia; ^3^ Department of Paediatrics Monash University Clayton VIC Australia; ^4^ Monash Newborn Monash Children's Hospital Clayton VIC Australia; ^5^ Department of Physiology Monash University Clayton VIC Australia; ^6^ Department of Physiology, Faculty of Health Sciences The University of Auckland Auckland New Zealand; ^7^ Monash Biomedical Imaging Monash University Clayton VIC Australia

**Keywords:** brain injury, brain volume, electrophysiology, FGR, IUGR, MRI, preterm

## Abstract

The last pregnancy trimester is critical for fetal brain development but is a vulnerable period if the pregnancy is compromised by fetal growth restriction (FGR). The impact of FGR on the maturational development of neuronal morphology is not known, however, studies in fetal sheep allow longitudinal analysis in a long gestation species. Here we compared hippocampal neuron dendritogenesis in FGR and control fetal sheep at three timepoints equivalent to the third trimester of pregnancy, complemented by magnetic resonance image for brain volume, and electrophysiology for synaptic function. We hypothesized that the trajectory of hippocampal neuronal dendrite outgrowth would be decreased in the growth‐restricted fetus, with implications for hippocampal volume, connectivity, and function. In control animals, total dendrite length increased with advancing gestation, but not in FGR, resulting in a significantly reduced trajectory of dendrite outgrowth in FGR fetuses for total length, branching, and complexity. Ex vivo electrophysiology analysis shows that paired‐pulse facilitation was reduced in FGR compared to controls for cornu ammonis 1 hippocampal outputs, reflecting synaptic dysfunction. Hippocampal brain‐derived neurotrophic factor density decreased over late gestation in FGR fetuses but not in controls. This study reveals that FGR is associated with a significant deviation in the trajectory of dendrite outgrowth of hippocampal neurons. Where dendrite length significantly increased over the third trimester of pregnancy in control brains, there was no corresponding increase over time in FGR brains, and the trajectory of dendrite outgrowth in FGR offspring was significantly reduced compared to controls. Reduced hippocampal dendritogenesis in FGR offspring has severe implications for the development of hippocampal connectivity and long‐term function.

## Graphical abstract

Hippocampal neuron dendritogenesis occurs rapidly in late fetal gestation and forms the foundation for connectivity and long term function. Fetal growth restriction caused by placental insufficiency is associated with reduced dendritogenesis in hippocampal neurons, resulting in a significant decrease in the trajectory of neuro‐development. This has significant implications for reduced hippocampal volume and poor memory function in children born growth restricted.

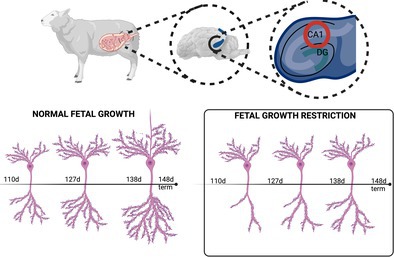

## 1 INTRODUCTION

Fetal growth restriction (FGR) is a common but serious pregnancy complication describing pathological low birth weight. In most cases, FGR occurs secondary to *placental insufficiency*, an umbrella term describing a dysfunctional placenta that fails to deliver adequate oxygen and nutrients to maintain fetal growth [1, 2, 3]. FGR is the second leading cause of perinatal mortality, behind prematurity, and for surviving infants, FGR poses a significant risk for morbidities of respiratory and cardiovascular function in the neonatal period [4, 5, 6, 7, 8]. Adverse outcomes associated with FGR are most profound in early‐onset FGR (diagnosed <32 weeks gestation), with severe placental disease in early‐onset FGR leads to preterm birth, fetal hypoxia, and a high likelihood of altered organ development [9]. Chronic in utero hypoxia that causes reduced body growth has adverse effects on all developing organ systems, including the brain [10, 11]. This is despite the presence of cerebrovascular compensation, which occurs as an adaptive response to hypoxia [12]. Cerebrovascular compensation (brain sparing) in human infants identified via reduced middle cerebral artery pulsatility index is a significant risk factor for neonatal death or brain injury [13]. A recently published systematic review of children born growth‐restricted has shown that cerebrovascular compensation during pregnancy is associated with worse cognitive function and lower intelligence quotient (IQ) in childhood [14].

Magnetic resonance imaging (MRI) demonstrates that total brain volume is reduced in infants with FGR, first evident in utero [15]. Soon after birth, total brain volume, cortical and hippocampal deficits, and cerebral disorganization are described [16, 17, 18], and persist at 1 year of age, with evidence of altered brain connectivity and reduced network efficiency [19, 20, 21]. Human MRI results are complemented by preclinical animal studies showing early evidence of white matter and glial cell injury in the FGR brain [22, 23, 24]. The hippocampus is a critical brain structure that regulates learning, memory, and emotional processing. Hippocampal neurons are present in the fetal brain by mid‐gestation, with hippocampal circuitry then laid down through the outgrowth of dendrites and synaptic connections to other cells [25, 26]. The effects of FGR on these critical neurodevelopmental processes are unknown, but MRI studies report reduced hippocampal volume in infants and children born with FGR [18, 21].

This study aimed to describe normal neuronal morphological development within the hippocampus by mapping dendrite outgrowth in appropriately grown fetuses over three gestational ages equivalent to the human third pregnancy trimester and to determine whether FGR was associated with altered neuro‐development. We utilized our sheep model of placental insufficiency which mimics early‐onset FGR with respect to the timing of placental dysfunction [22, 27], and collected brain tissue during the last trimester of pregnancy from FGR and control fetuses at 110, 127, and 138 days (148 days, full gestation), broadly comparable to human pregnancy between 28 weeks' gestation to term‐equivalent age [28]. We quantified neuronal cytoarchitecture in hippocampal cornu ammonis 1 (CA1) pyramidal neurons using Golgi‐Cox staining and immunohistochemical techniques. We complemented cellular‐level analysis with brain MRI at the mid‐timepoint (127 days) for brain volume assessment and hippocampal slice electrophysiology for synaptic function. We hypothesized that the trajectory of dendrite arborization would be reduced in FGR hippocampal neurons compared to controls, with implications for hippocampal volume and neuronal connectivity and function.

## 2 MATERIALS AND METHODS

### 2.1 Study approval

All animal experiments were approved by the Monash Medical Centre Animal Ethics Committee (MMCA/2016/62 & MMCA2016/24) and conducted in accordance with the National Health and Medical Research Council (NHMRC) of Australia, Australian Code for the Care and Use of Animals for Scientific Purposes, informed by the ARRIVE guidelines for the reporting of animal research.

### 2.2 Animal surgery

The methodology for inducing early‐onset FGR in fetal sheep has been described in detail previously [22, 27]. Briefly, pregnant cross‐bred ewes carrying twins underwent sterile surgery at 88–90 days gestational age (60% gestation) and under general anesthesia (1%–2.5% isoflurane in oxygen, Bomac, New South Wales, Australia), single umbilical artery ligation was undertaken to induce FGR in one fetus, while the umbilical cord of the twin control fetus was handled only. A catheter was inserted into the maternal jugular vein for administration of post‐operative antibiotics and euthanasia at the completion of the experiment. At the cessation of the surgical procedure and after withdrawal of anesthesia, the ewe was monitored closely and given oral Panadol (1 g), iv ampicillin (1 g) and engemycin (500 mg), for 3 days. Ewes were housed together in individual pens in a 12‐h light–dark cycle with free access to food and water and monitored daily.

### 2.3 Longitudinal study

#### 2.3.1 Experimental design

Ewes were randomly allocated to one of three cohorts that dictated the timing of postmortem. Groups were (i) early preterm (110 days), (ii) preterm (127 days), and (iii) term‐equivalent brain development (138 days). Control animals were used as a baseline compared to FGR animals. At each timepoint, ewes and fetuses were euthanized via maternal injection of pentobarbitone (Lethobarb; Virbac, Peakhurst, Australia).

At postmortem, the fetus was weighed, and the fetal brain was removed, weighed, and divided sagitally into left and right hemispheres. The right hemisphere was sectioned coronally into 5 mm slices and immersion fixed in 10% Neutral Buffered Formalin (Trajan Scientific, #NBF‐10L, Australia) for 5 days. This was followed by standard paraffin embedding for immunohistochemistry, prior to sectioning with a microtome at a thickness of 10 μm at the mid‐thalamic level, containing the hippocampus (corresponding to section 1120 in the Sheep Ovis Aries atlas, Michigan State University). Sections were mounted on SuperFrost Plus slides (Thermoscientific, USA). The left hemisphere was sectioned coronally at 1–1.5 cm thickness and processed immediately with the FD‐Rapid GolgiStain kit (FD NeuroTechnologies, Inc., PK401 Cell Systems Biology) [29]. Sections were cut on a vibratome (VT1200S, Leica) at 100 μm.

#### 2.3.2 Hippocampal CA1 neuronal morphology

Hippocampal CA1 pyramidal neurons were analyzed. Apical and basal dendritic arbors were quantified separately, with all analyses conducted on coded slides for experimental blinding. We followed a pre‐determined cell selection criterion for pyramidal CA1 neurons, which included (i) pyramidal neuron with a triangular‐shaped soma located in the stratum pyramidale; asymmetric dendritic length with apical dendrites projecting to the stratum radiatum and basal dendrites to the stratum oriens; (ii) no dendritic overlap; and (iii) complete visibility of apical and/or basal dendrites. Exclusion criteria were (i) dendritic overlap; (ii) truncated neurons; and (iii) neurons with incomplete dye transport [29]. An average of six pyramidal neurons per animal per group was used for apical analysis and seven pyramidal neurons per animal per group for basal analysis, with apical and basal dendrites assessed in the same neuron. In total, the number of reconstructed neurons used for apical dendrite analysis were: 110 days—control *n* = 29, FGR *n* = 28; 127 days—control *n* = 63, FGR *n* = 43; 138 days—control *n* = 53, FGR *n* = 19, and for basal dendrites: 110 days—control *n* = 31, FGR *n* = 31; 127 days—control *n* = 70, FGR *n* = 55; 138 days—control *n* = 64, FGR *n* = 27.

Cell imaging and analysis followed published procedures, using the Imaris Filament tracing tool and Imaris XT [29]. Total (basal and apical combined), basal, and apical dendritic length (μm) and dendrite branch number were quantified. Sholl analysis of ring intersections was performed to quantify basal and apical dendrites as a measure of cell complexity. Sholl analysis quantifies the number of dendritic intersections from the cell soma, at set distance intervals from the soma; for apical dendrites, this distance was set at 20 μm intervals, and for basal dendrites, this was at 5 μm intervals. Dendritic spines were quantified by total number and density (spines/10 μm distance) individually for basal and apical dendrites.

#### 2.3.3 Immunohistochemical staining

Immunohistochemical staining was used to identify neuronal nuclei (neuronal nuclear protein, NeuN, Millipore/Sigma‐Aldrich, #MAB377), synapses (Synapsin I, Syp‐1, Abcam, #ab64581) and brain‐derived neurotrophic factor (BDNF), (Abcam, #ab108319) [22, 30, 31] on hippocampal CA1 cells. Antibodies were diluted in PBS and all sections were treated with a corresponding biotinylated secondary antibody (1:200; NeuN and Syp‐1: Vector Labs #BA‐9200 or #BA‐1000, Newark, CA, BDNF: goat anti‐rabbit IgG conjugated with Alexa Fluor 488, Thermo Fisher Scientific, #A11008). Immunolabeling was visualized using 3,3‐diaminobenzidine (DAB; Pierce Biotechnology, Rockford, IL) and positive and negative control sections were examined. Sections were viewed at 400× magnification using a light microscope (Olympus BX‐41, Japan). Analysis was undertaken on three images per slide in duplicate, resulting in 6 images per animal, with assessors blinded to a group using coded slides. Positive immunohistochemical staining for the densitometric area of Syp‐1 and BDNF was acquired using CellProfiler v4.0.7 and ImageJ v.2.1.0 respectively, after setting a background threshold for all images for each antibody. NeuN‐positive cells were manually counted using ImageJ.

### 2.4 MRI and ex vivo electrophysiology study

#### 2.4.1 Experimental design

On a separate cohort at 127 days (corresponding to ~32–34 weeks gestation in humans, which is a high‐risk time for preterm birth associated with early‐onset FGR [32]), control (*n* = 12) and FGR (*n* = 9) fetuses were delivered by cesarean section and lambs immediately transferred to an infant warmer, intubated and ventilated at a target of 5–7 mL/kg (Babylog 8000+, Dräger, Lüberk, Germany), and administered prophylactic surfactant (100 mg/kg, Curosurf, Chiesi, Italy) as described previously [33]. Umbilical artery and vein catheters were inserted, and a pulse oximeter probe (Masimo, CA, USA) was placed on the lamb's tail for SpO_2_ measurement. Prior to MRI, lambs were lightly sedated with continuous iv infusion of Alfaxalone (3 mg/kg/min; Jurox, NSW, Australia). When stable, lambs were transferred to a 3.0T MRI scanner (Siemens Skyra, Erlangen, Germany) with the lamb's head placed in a 15‐channel transmit/ knee coil, in a supine position with continued ventilation using MR compatible ventilator (BabyPac MR, Pneupac, Smiths Medical, Kent, UK). The specifics of the sequence parameters have been described previously [33]. Briefly, T1‐ and T2‐weighted images were acquired. A 3D T2‐weighted image was acquired using a fast spin echo sequence with the following parameters: 0.5 × 0.5 × 0.5 mm^3^ isotropic voxels, 384 × 384 acquisition matrix, Repetition time/Echo time (TR/TE) = 1000/130 ms, NEX = 2, and echo train length = 64. A T1‐weighted image was generated using a spin‐echo sequence with inversion recovery. The parameters were as follows: TR/TE = 1440/3.92 ms, inversion time = 900 ms, NEX = 1, 0.8 × 0.8 × 0.8 mm^3^ isotropic voxels and acquisition matrix = 256 × 256. Once the MRI was complete, lambs were euthanased (pentobarbitone as above) and immediately cardiac‐perfused with cold *N*‐methyl‐d‐glucamine‐artificial cerebrospinal fluid (aCSF), the brains were quickly removed and sections of the brain containing the hippocampus were sliced in *N*‐methyl‐d‐glucamine‐aCSF solution at a thickness of 400 μm using a ceramic blade in a Campden Instruments (England) Integraslice 7550 MM.

#### 2.4.2 Ex vivo electrophysiology

Hippocampal slices were recovered in HEPES‐aCSF at room temperature, after which they were placed in a recording chamber on the stage of an upright microscope (Leica DMLFS) and superfused with normal aCSF at 32°C at a rate of 3 mL/min. Extracellular responses were recorded from the *stratum radiatum* of CA1 following stimulation of the Schaffer collaterals. The recording electrode was a glass patch‐pipette‐like electrode filled with normal aCSF, while the stimulating electrode was an Ag/AgCl wire inside a glass pipette. The electrodes were placed 300 μm apart and 200 μm from the *stratum pyramidale*. The signals were fed into an Axoclamp 2 amplifier, digitized at 10 kHz with a Digidata 1332A, and recorded and analyzed with pClamp 10 software (Axon Instruments, CA, USA).

For paired‐pulse facilitation (PPF), field excitatory postsynaptic potentials were recorded in response to stimuli applied with interpulse intervals of 500, 300, 160, 70, and 25 ms (eight responses averaged for each interval). The PPF ratio was determined as the ratio of the peak amplitudes. Input/output relationships were determined by recording the responses to stimuli of 0.5–10.0 V (dial setting; five responses averaged for each stimulus strength).

#### 2.4.3 MRI volume analysis

FSL‐brain extraction tool was used to extract the brain and create a brain mask. All images were inspected, and the extracted brains were corrected manually for accuracy of coregistration and total volume measurements. Total white and grey matter volumes were calculated from T1 images using the FAST segmentation tool in FSL (the FMRIB Software Library; https://fsl.fmrib.ox.ac.uk/fsl/fslwiki). Partial volume maps extracted from FAST were corrected manually using FreeSurfer (https://surfer.nmr.mgh.harvard.edu/) where needed. Total white matter and grey matter volumes were calculated using FSL. For region of interest (ROI) volume analysis, T2 images were denoised and then unbiased using ANTs (https://picsl.upenn.edu/software/ants/). A sheep brain template and ATLAS (NITRC Turone Sheep Brain Template) were used for ROI extraction. The orientation of the T2 images was corrected using FSL to align with the orientation of the template and T2 images were coregistered to the sheep brain template using the ANT coregistration tool. The resulting transformation matrix was used to reverse‐transform the ATLAS labels to the T2 image. ROIs evaluated included the thalamus, hippocampus, cerebellum, and internal capsule. All ROIs were inspected to ensure that they covered the correct region in all images. The analysis was undertaken by one experienced rater (TS) who was blinded to the animal identifier.

### 2.5 Statistical analyses

GraphPad Prism (version 10.2.1, GraphPad software, MA, USA) was used for statistical analyses. Data are presented as mean ± SEM. In the first instance, we assessed gestational age changes in body weight, brain: body weight, dendritic length, dendritic branch number, and immunohistochemical analyses separately for each parameter in the control and FGR groups at 110, 127, and 138 days using one‐way ANOVA. This was followed by a two‐way ANOVA on the same outcomes to determine the effects of the independent variables of time (110, 127, and 138 days) and growth status (control vs. FGR). Electrophysiology data were assessed using two‐way repeated measures. Where a significant effect of the independent variables was shown, post hoc comparisons were made using Tukey's multiple comparisons for normally distributed data. Non‐parametric data were analyzed using Kruskal–Wallis ANOVA on ranks with Dunn's method. Sholl analysis of ring intersections was performed for all reconstructed cells and each gestational age was analyzed separately using repeated‐measures two‐way ANOVA with growth status (control vs. FGR) and radius set as variables. An unpaired *t*‐test was used to measure differences between areas under the curve for Sholl analysis. The trajectory of change was calculated across timepoints separately within the control and FGR groups using Pearson's correlation, and a comparison between groups was assessed by determining whether the slope or intercept of the lines was different between the control and FGR cohorts. Significance was accepted at *p* < 0.05.

## 3 RESULTS

### 3.1 Longitudinal study

#### 3.1.1 Lamb biometry

**FIGURE 1:**
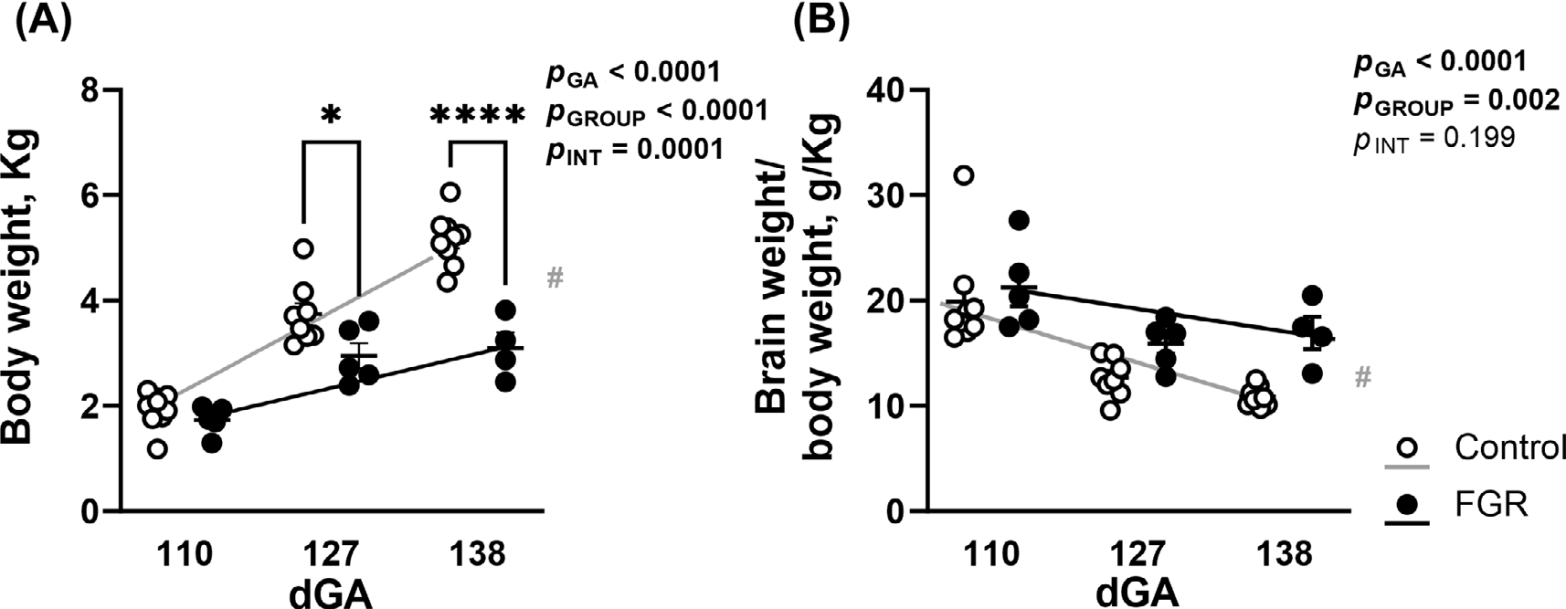
Fetal body weight and brain:body weight ratio across gestation. Body weight (A) and brain weight were corrected for body weight (B) in control (white) and fetal growth restriction (FGR; black) fetuses at 110, 127, and 138 days gestation (dGA). Data are mean ± SEM *N* = 4–9 per group. Two‐way ANOVA with gestational age and group (control vs. FGR) as variables with Šídák's multiple comparisons test. ****p < 0.0001, *p < 0.05 between groups (control vs. FGR). Linear regression analysis is overlaid for control (grey line) and FGR (black line) fetuses, # indicates significantly different slope or intercept between groups (control vs. FGR).

A total of 40 fetal sheep were included in the longitudinal study across three gestational ages (110–112 days: control *n* = 9 [3M:6F], FGR *n* = 5 [2M:3F]; 124–127 days: control *n* = 8 [5M:3F], FGR *n* = 5 [3M:2F]; 138–139 days: control *n* = 9 [4M:5F], FGR *n* = 4 [1M:3F]). There were eight fetal losses in the FGR cohort (two in the 110 days, three in the 127 days, and three in the 138 days cohort), with these data excluded from analysis. Unexpectedly, we had two triplet pregnancies, and the unoperated fetuses were allocated to a control group. One‐way ANOVA of the body weight of control fetal sheep demonstrated a significant increase over late gestation (*p* < 0.0001), with a 2.7‐fold increase in body weight between 110 and 138 days (*p* < 0.0001; Figure 1A). By comparison, FGR fetuses exhibited a 1.8‐fold increase in body weight over the same period (*p* = 0.002), but with no increase in body weight between 127 and 138 days (*p* = 0.889). Two‐way ANOVA showed that the body weight of FGR fetuses was significantly less than controls (*p* < 0.0001) with a gestational increase in body weight (*p* < 0.0001). A significant interaction was observed (*p* < 0.0001), and post hoc analysis showed that FGR fetuses weighed less than control fetuses at 127 days (*p* = 0.019) and 138 days (*p* < 0.0001). Brain weight corrected for body weight is shown in Figure 1B, with a significant effect of FGR (*p* = 0.002) and gestational age (*p* < 0.0001). Linear regression showed an increasing trajectory of body weight in control (*r* ^2^ = 0.895, *p* < 0.0001) and FGR (*r* ^2^ = 0.567, *p* = 0.002) fetuses over late gestation, but the slope was reduced in FGR animals (*p* < 0.0001).

#### 3.1.2 Arborization trajectory of control and FGR hippocampal neurons

**FIGURE 2:**
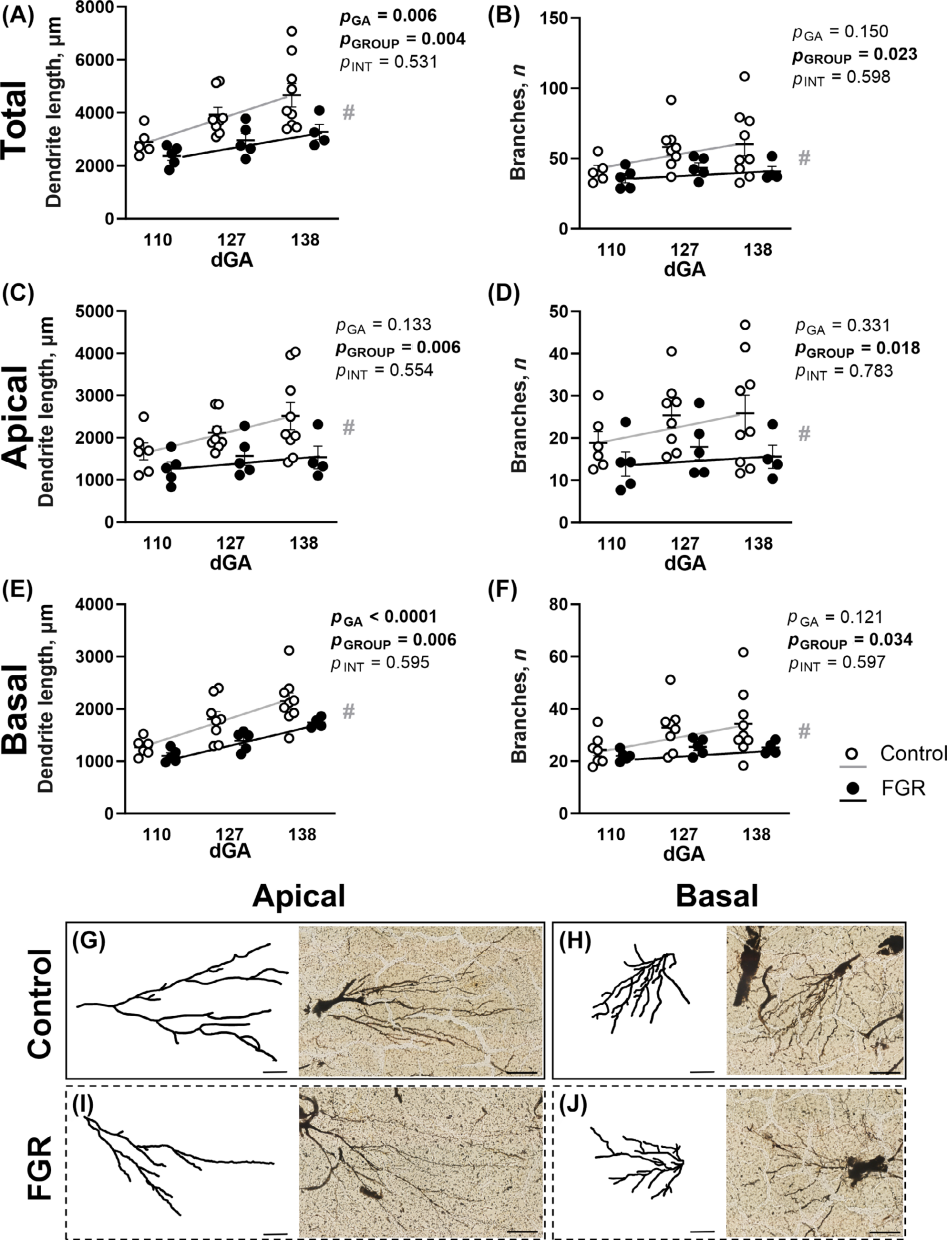
Hippocampal neuron analysis of dendrite length and branch number. Golgi‐stained analysis for total summated dendrite length (A), total summated branch number (B), apical dendrite length (C), apical branch number (D), basal dendrite length (E) and basal branch number (F) in control (white) and fetal growth restriction (FGR; black) fetuses at 110, 127, and 138 days gestation (dGA). Representative dendrite morphology using mean values for dendrite length and branching at 127 dGA in control (G, H) and FGR (I, J) fetuses, shown for both apical and basal arbors with corresponding Golgi‐stained sections. Scale bar = 50 μm. Data are mean ± SEM *N* = 4–9 per group. Two‐way ANOVA with gestational age and group (control vs. FGR) as variables with Šídák's multiple comparisons test. Linear regression analysis is overlaid for control (grey line) and FGR (black line) fetuses, # indicates significantly different intercepts between groups (control vs. FGR). Scale bar = 50 μm.

We assessed the normal developmental profile of hippocampal neurons in healthy control fetal brains to map the trajectory of dendritogenesis in late pregnancy. In controls, total dendritic length (i.e., combined apical and basal dendrites) increased with advancing gestation, with a 1.6‐fold increase between 110 and 138 days gestation (*p* = 0.02; Figure 2A). By contrast, FGR animals did not show a significant increase in total dendritic length (*p* = 0.07) or total branch numbers (*p* = 0.30) from 110 to 138 days gestation. Two‐way ANOVA found a significant difference in the total dendritic length between the groups (*p* = 0.004) and over time (*p* = 0.006), with no interaction (*p* = 0.531). Two‐way ANOVA for total branch numbers showed fewer dendritic branches in FGR animals (*p* = 0.023; Figure 2B). The intercept of regression lines was significantly different between control and FGR for both total dendrite length (*p* = 0.002) and total dendritic branch numbers (*p* = 0.017).

A separate analysis of apical and basal dendritic length and branching showed that both the apical and basal dendrites were susceptible to altered development in FGR (Figure 2C–F). Specifically, there was a reduction in both the length and number of branches of apical dendrites in FGR animals (*p* < 0.05; Figure 2C,D), while the trajectories for both dendrite length (*p* = 0.005) and branch numbers (*p* = 0.015) were different in control and FGR animals over time. For basal dendrites, both dendritic length and the trajectory of dendritic length were reduced in FGR (*p* = 0.006 and *p* = 0.004, respectively; Figure 2E), as was the number of branches and the trajectory of dendritic branch numbers (*p* = 0.034 and *p* = 0.032, respectively; Figure 2E,F). Representative images for Golgi‐stained apical (Figure 2G,I) and basal (Figure 2H,J) dendritic arbors, and skeletonized traced arbors used for quantification, are presented for the mean values obtained in control and FGR animals from the 127 days gestation group. Note the propensity for simplification of FGR dendritic arbors with one or two long dendrites that lacked branches, and short and stubby dendrite outgrowth.

**FIGURE 3:**
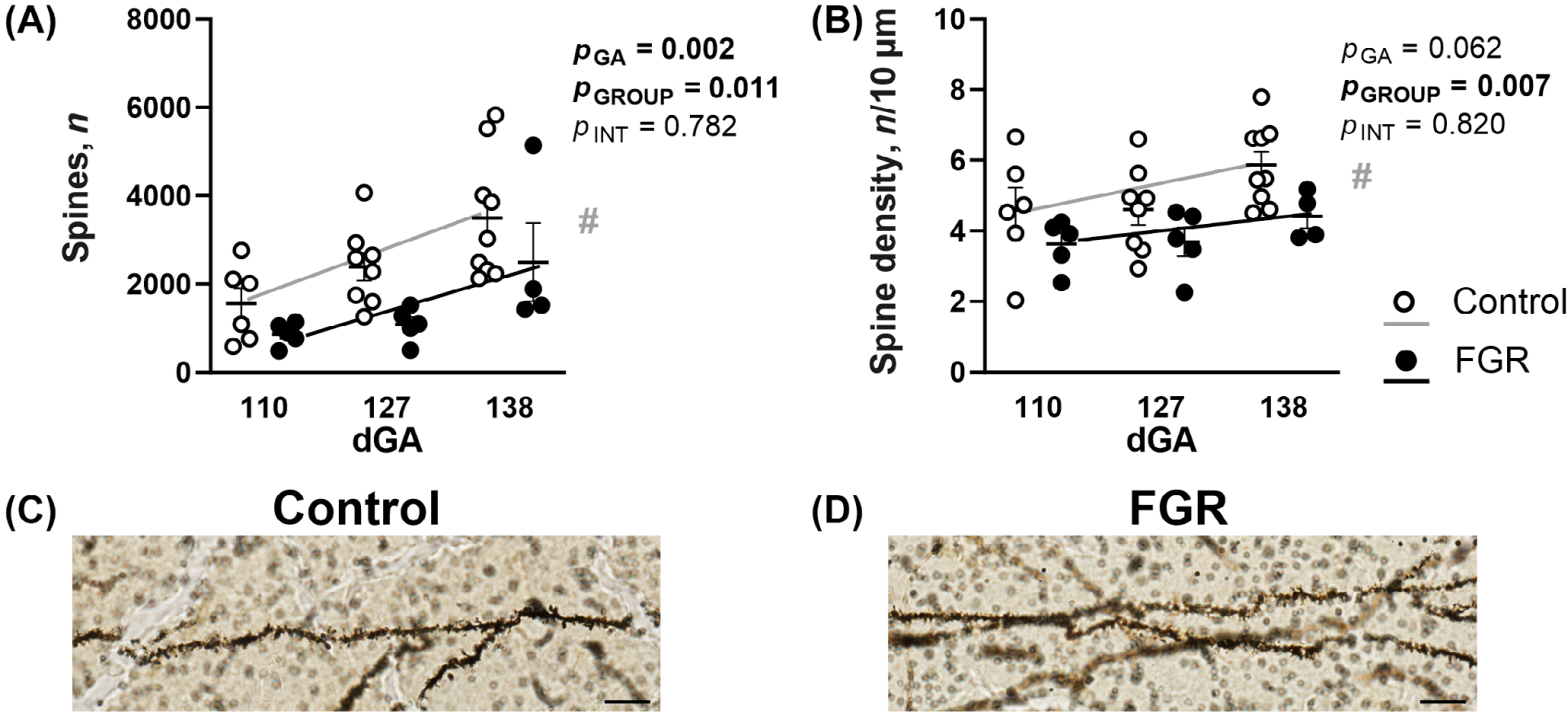
Hippocampal neuron analysis of spines. Golgi‐stained analysis for total summated spine number (A) and total spine density (B) in control (white) and fetal growth restriction (FGR; black) fetuses at 110, 127, and 138 days gestation (dGA) with representative images of spines from control (C) and FGR (D) at 127 dGA. Data are mean ± SEM *N* = 4–9 per group. Two‐way ANOVA with gestational age and group (control vs. FGR) as variables with Šídák's multiple comparisons test. Linear regression analysis is overlaid for control (grey line) and FGR (black line) fetuses, # indicates significantly different intercepts between groups (control vs. FGR). Scale bar = 10 μm.

Dendritic spines form the principal sites for excitatory input on neurons. The total number and density (spines/10 μm) of dendritic spines were quantified across the control and FGR groups over the course of late gestation. We found a significant reduction in both the total number (*p* = 0.011; Figure 3A) and density (*p* = 0.007; Figure 3B) of dendritic spines in the FGR cohort. Two‐way ANOVA revealed that total spine number and total spine density were both reduced in FGR animals (*p* = 0.011 and *p* = 0.007, respectively). Further, regression analysis showed different developmental trajectories for total spine number (*p* = 0.008) and total spine density (*p* = 0.007) between the groups. Representative high‐power images used for spine analysis on Golgi‐stained dendrites are presented for control (Figure 3C) and an FGR (Figure 3D) dendrite.

#### 3.1.3 Sholl analysis for neuronal complexity

**FIGURE 4:**
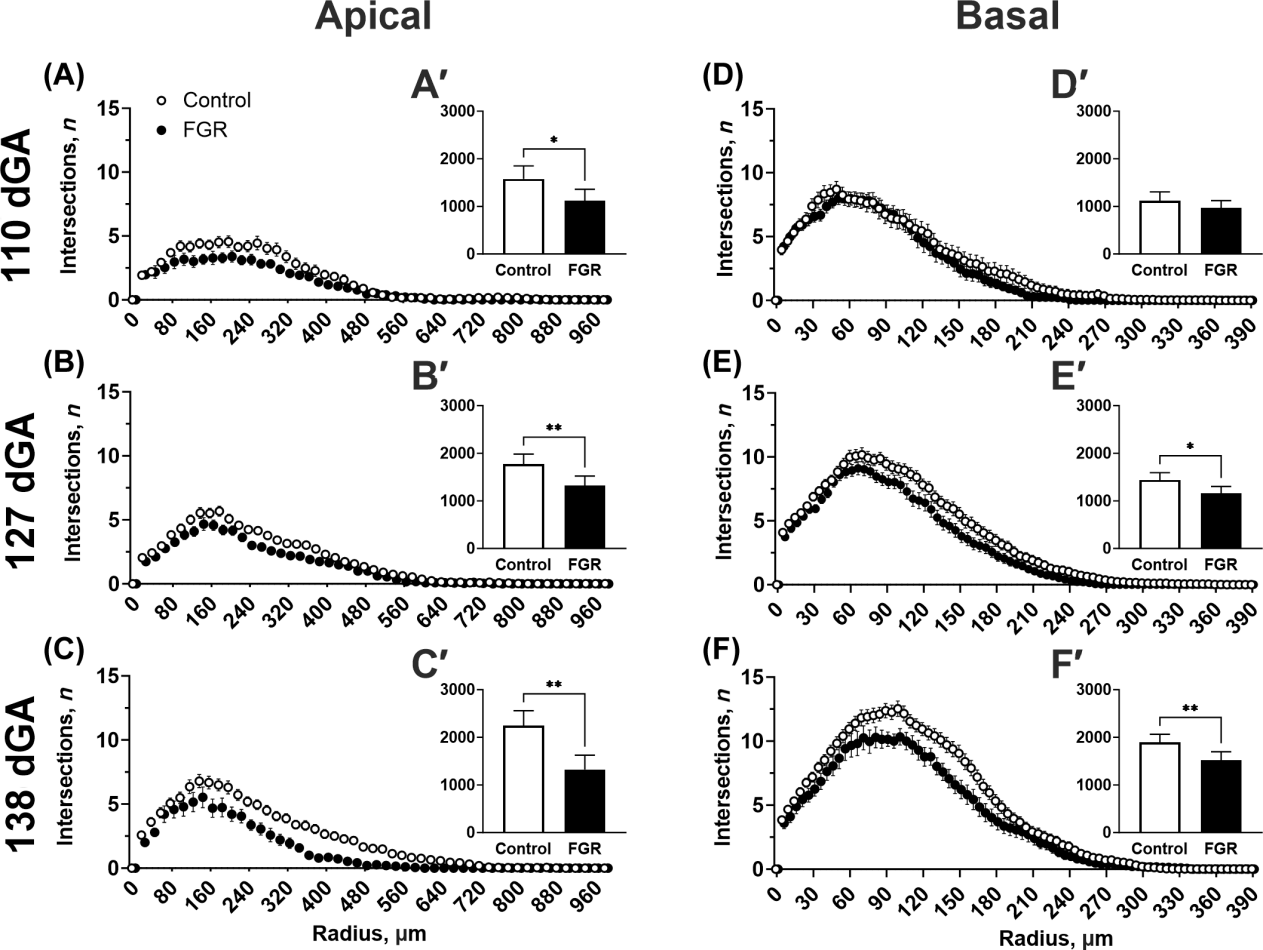
Hippocampal neuron analysis of dendrite complexity. Sholl analysis (A–F) and total area under the curve (A′–F′) for control (white) and fetal growth restriction (FGR; black) fetuses at 110, 127, and 138 days gestation (dGA). Data are mean ± SEM *N* = 4–9 per group. Two‐tailed unpaired *t*‐test **p < 0.01, *p < 0.05 between groups (control vs. FGR).

We next performed Sholl analysis to quantify morphological differences in arborization complexity by counting the number of dendritic intersections and branches at fixed distances from the cell soma (Figure 4). Two‐way ANOVA showed a significantly decreased number of intersections in FGR fetuses compared to control fetuses for apical dendrites at 110 days (*p* = 0.014), 127 days (*p* = 0.004) and 138 days (*p* = 0.002), and for basal dendrites at 127 days (*p* = 0.011) and 138 days (*p* = 0.009). These corresponded to the overall difference in complexity (area under the curve) for apical dendrites at 110 days (*p* = 0.01), 127 days (*p* = 0.004), and 138 days (*p* = 0.002) in the FGR group compared with controls. Similarly, there was a significant difference in basal dendritic complexity at 127 days (*p* = 0.01) and 138 days (*p* = 0.01), but not at 110 days (*p* = 0.23), in the FGR cohort compared with controls.

#### 3.1.4 Immunohistochemical analyses

**FIGURE 5:**
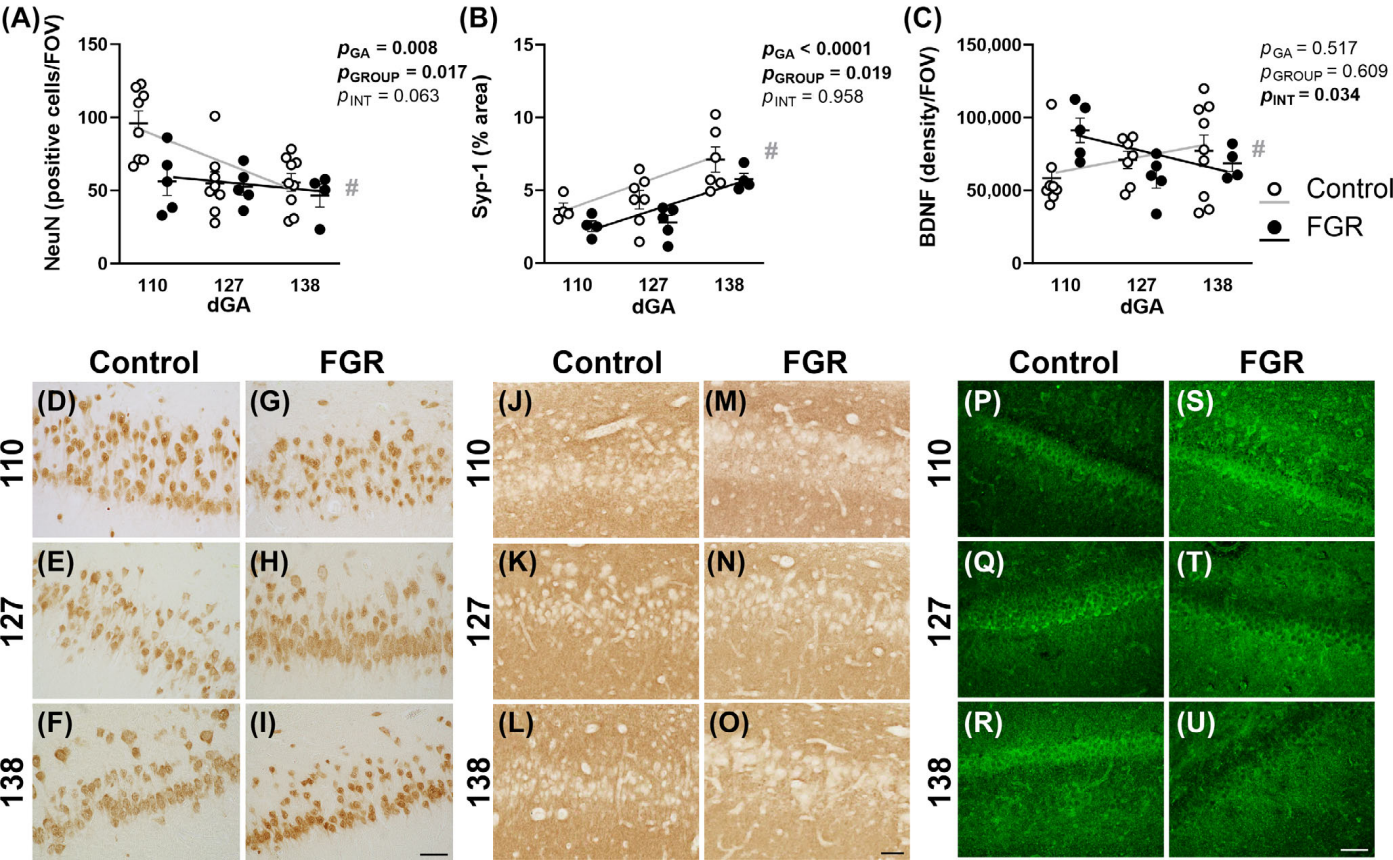
Immunohistochemical analysis. Neuronal cell counts (NeuN, A), synapsin‐1 (Syp‐1) % area positive staining (B) and brain‐derived neurotrophic factor (BDNF) staining density (C) in the hippocampus for control (white) and fetal growth restriction (FGR black) fetuses at 110, 127, and 138 days gestation (dGA) with representative photomicrographs of NeuN (D–I), Syp‐1 (J–O) and BDNF (P–U) staining in control and FGR fetuses at 110, 127, and 138 dGA. Data are mean ± SEM. *N* = 4–9 per group. Two‐way repeated measures ANOVA with gestational age and group (control vs. FGR) as variables with Šídák's multiple comparisons test. Linear regression analysis is overlaid for control (grey line) and FGR (black line) fetuses, # indicates significantly different slope or intercept between groups (control vs. FGR). Scale bar = 50 μm.

In control animals, immunohistochemistry for NeuN+ neurons showed a decrease in CA1 hippocampal neuronal density from 110 to 138 days gestation (*p* = 0.0009; Figure 5A,D–F), likely reflecting an increasing sparsity of neuronal cell bodies with increasing hippocampal area. By contrast, FGR animals showed no change in CA1 neuron density from 110 to 138 days gestation (*p* = 0.71; Figure 5A,G–I). Two‐way ANOVA for assessment of group and time effect revealed a reduced number of neurons across gestation (*p* = 0.008) as well as a reduction in FGR animals (*p* = 0.017), which is accounted for by a lower neuron count in FGR animals at 110 days (*p* = 0.023) that did not persist at 138 days (*p* > 0.99). The density of Syp‐1 increased over gestation in both controls (*p* = 0.013) and FGR animals (*p* = 0.0008), but with an overall deficit in FGR animals (*p* = 0.019) and a reduced trajectory of Syp‐1 in FGR compared with control animals (*p* = 0.029; Figure 5B,J–O). Given that endogenous neurotrophic factors within the developing brain, particularly BDNF, are potent regulators of dendritic and synaptic development [34, 35], we also assessed BDNF abundance in the hippocampus. In control animals, there was no change in BDNF over gestation (*p* = 0.32; Figure 5C,P–R). Contrastingly, FGR animals showed decreased BDNF staining across gestation (*p* = 0.021, Figure 5C,S–U), which was largely due to reduced BDNF at 127 days compared with 110 days gestation (*p* = 0.019). The slopes of BDNF immunostaining across gestation in control and FGR hippocampus demonstrated diametric profiles, with increasing abundance in control animals compared with decreasing abundance in FGR animals (*p* = 0.018).

### 3.2 MRI and ex vivo electrophysiology study

#### 3.2.1 Ex vivo electrophysiology

**FIGURE 6:**
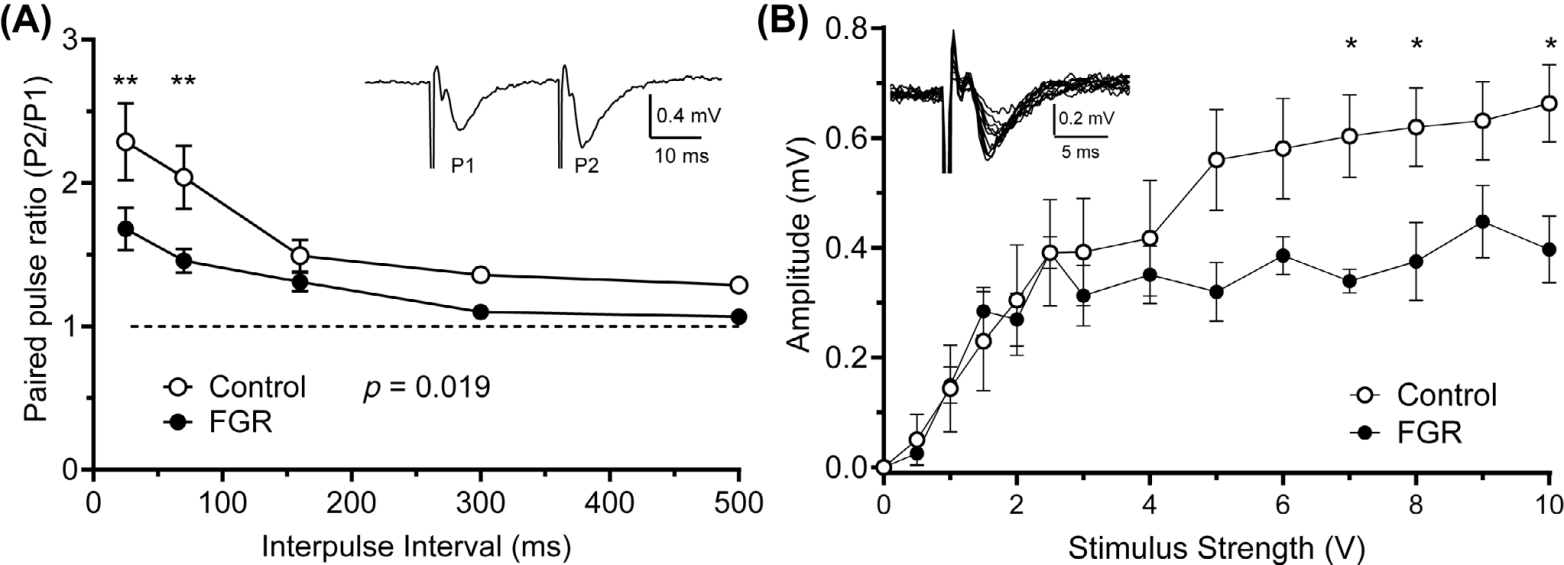
Hippocampal electrophysiology. Paired‐pulse facilitation (PPF) in hippocampal cornu ammonis 1 region for interpulse intervals of 25–500 ms (A) with inset example trace data of PPF with an interpulse interval of 25 ms. Input/output relationships (B) for control (white) and fetal growth restriction (FGR; black) fetuses at 127 days gestation with inset example of data traces for the responses evoked by increasing stimulus strengths. Data are mean ± SEM. *N* = 6 control and *n* = 5 FGR. Two‐way repeated measures ANOVA with Tukey multiple comparisons test. **p < 0.01, *p < 0.05 between groups (control vs. FGR).

Next, we examined functional changes in hippocampal neuronal circuits using hippocampal brain slices collected from control and FGR lambs at 127 days gestation. Paired stimulating pulses were applied to the Schaffer collaterals and the resulting field excitatory postsynaptic potentials were recorded from the *stratum radiatum*. For interpulse intervals ≥160 ms, the paired‐pulse ratios were similar between the groups (i.e., close to 1; Figure 6A)—this is not surprising as values of 1 are expected for long intervals. By contrast, for shorter intervals, there was a significant decrease (*p* = 0.016) in the degree of facilitation in the CA1 region from FGR lambs compared with controls. For the input/output relationships in the CA1 region (Figure 6B), there was an increasing response with increasing stimulus in control animals, which plateaued at higher stimulus strengths. At these higher stimulus strengths, the maximal response recorded from the FGR group was reduced compared with controls (*p* < 0.05 for stimulus strengths 7, 8, and 10 V).

#### 3.2.2 Brain volume

**FIGURE 7:**
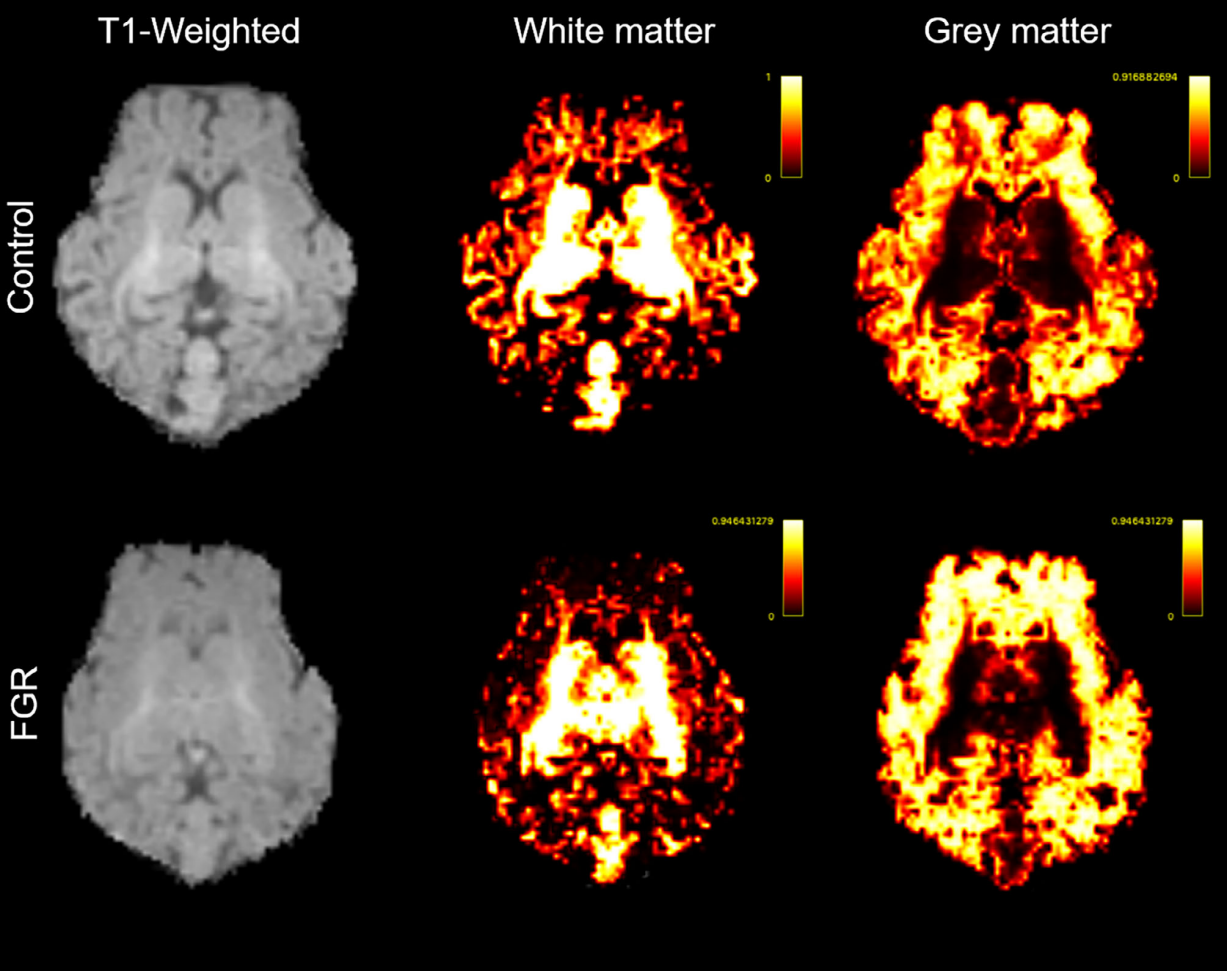
Magnetic resonance imaging brain volume. Non‐binary partial volume images for each class in a representative control brain (top panels) and fetal growth restriction brain (bottom panels) at 127 days gestation, showing white matter and grey matter voxel‐based tissue volumes. Each voxel contains a value in the range 0–1 (as shown in the scale) that represents the tissue classification present in that voxel.

The whole brain and regions of interest volumes are shown in Table 1, with representative images shown in Figure 7. Whole brain volume was reduced by 9.5% in FGR animals (*p* = 0.012), which was largely due to a reduction in white matter (*p* = 0.003) but not grey matter (*p* = 0.191) volumes. The volume of the left internal capsule was reduced in FGR animals (*p* = 0.027). Total and left hippocampal volumes were non‐significantly reduced (*p* = 0.354 and *p* = 0.180, respectively) compared with controls. Interestingly, of the grey matter regions of interest examined, the left hippocampus to left thalamus ratio was significantly reduced in FGR animals compared with controls (*p* = 0.032), indicating that within grey matter regions, the hippocampus may be preferentially susceptible to volume loss in FGR.

**TABLE 1:** Magnetic resonance imaging‐derived brain and regional volumes in control and fetal growth restriction (FGR) fetuses at 127 days gestation.

Regional volume (mm^3^)	Control (*n* = 12)	FGR (*n* = 8)	*p*‐value
Whole brain	59,180 ± 1368	**53,555 ± 1456** *	**0.012**
White matter	23,005 ± 536.4	**20,123 ± 694.3** *	**0.003**
Grey matter	24,107 ± 572.9	22,852 ± 749.8	0.191
White:grey matter	0.96 ± 0.02	0.89 ± 0.05	0.179
CSF	12,068 ± 532.3	10,580 ± 709.0	0.103
Total hippocampus	542.6 ± 14.3	519.8 ± 20.2	0.354
L hippocampus	259.1 ± 6.2	243.4 ± 10.2	0.180
R hippocampus	283.5 ± 8.5	276.4 ± 10.5	0.608
L cortex	1679 ± 44.9	1718 ± 79.2	0.647
R cortex	1519 ± 40.3	1500 ± 82.4	0.820
L internal capsule	105.1 ± 3.8	**91.5 ± 3.8** *	**0.027**
R internal capsule	115.1 ± 3.7	110.8 ± 4.4	0.472
L thalamus	784.6 ± 19.2	794.1 ± 25.9	0.766
R thalamus	750.3 ± 21.5	748.2 ± 27.6	0.953
L hippocampus:cortex	0.155 ± 0.004	0.142 ± 0.005	0.062
R hippocampus:cortex	0.187 ± 0.006	0.187 ± 0.010	0.983
L hippocampus:thalamus	0.331 ± 0.005	**0.307 ± 0.010** *	**0.032**
R hippocampus:thalamus	0.378 ± 0.006	0.372 ± 0.016	0.675

Abbreviations: CSF, cerebrospinal fluid; L, left; R, right.

* *p* < 0.05, and represented in bold text.

## 4 DISCUSSION

This study provides compelling evidence that FGR is associated with a reduced trajectory of neuronal maturation and function in the developing hippocampus. Deviation from normal neurodevelopment in FGR occurs in utero over the course of the third trimester of pregnancy, underpinned at the cellular level by reduced neuronal length and branching and decreased overall complexity of dendritic arbors. Hippocampal neurite outgrowth in the normal healthy brain significantly increased over the course of the third trimester of pregnancy with increasing dendrite length, branching and complexity, together with increasing trajectory of spine and synaptophysin density. In contrast, in the hippocampus of FGR offspring, there was no increase in neuron dendrite length, density or spine number over late gestation. This is the first study to show that the trajectory of hippocampal neuron dendritogenesis is significantly impaired in the FGR brain compared to the control. This is an important finding, because it is the pattern of dendritic arborization that determines neuronal communications with other cells [36]. MR imaging showed that brain volume was reduced in the FGR cohort, and while there was no significant deficit in hippocampal volume in FGR brains per se at the mid‐gestational age timepoint (127 days), results demonstrate that the hippocampus was more susceptible to volume deficit than the thalamus. The morphometric deficits in CA1 arborization in FGR fetuses were associated with an altered capacity for synaptic connectivity within the hippocampal circuitry, with resultant disruption to circuit function.

This study assessed the normal trajectory of neurite growth in healthy hippocampal neurons over the final trimester of sheep pregnancy, demonstrating a 60% increase in total dendritic length of combined apical and basal arbors between 0.7 and 0.9 of gestation, broadly aligned with the last trimester of human brain development [28]. The increasing structural morphology of CA1 neurons via neurite outgrowth (i.e., axons, dendrites, and spines), is fundamental to laying the circuitry for cell‐to‐cell connections within and external to the hippocampus [26], underpinning cognitive and learning capabilities. In the human fetal brain, neurons are present within the hippocampus with a basic structure that resembles the adult form from about mid‐gestation [37]. There is then a linear volume increase of the hippocampal structure toward term gestation [38], due to rapid dendrite outgrowth and arborization of pyramidal neurons [26]. The current study maps the cellular basis for this normal profile, with an increasing trajectory of dendritic outgrowth, arborization complexity, and spine development in the healthy hippocampus in the final stages of pregnancy. In comparison, the maturational profile of hippocampal arborization was significantly blunted in FGR fetuses.

The organizational pattern of dendritic arbors was simplified in FGR neurons. In comparison to the dynamic nature of dendritogenesis and the increasing complexity of arbors in healthy brains, neuronal morphology was profoundly affected in animals with FGR. Total dendrite length was decreased in neurons of the FGR brain compared to control brains, concomitant with a reduced number of dendritic branches and spines. These deficits in the outgrowth of neuronal processes are compounded over the course of late pregnancy, to cause a significant decline in the trajectory of dendritic outgrowth and overall dendritic complexity of hippocampal neurons in FGR offspring, with neither total dendrite length nor branch number increasing between 110 and 138 days gestation within the FGR brain. Sholl analysis showed that in apical and basal dendritic arbors, total neuronal complexity was reduced in FGR animals. This resulted in a modified dendritic arborization pattern in FGR, in which the maximum number of branches (intersections) was achieved closer to the neuronal soma and with fewer branches that were shorter. A previous study in guinea pig fetuses exposed to chronic placental insufficiency showed a decrease in dendrite length and branching on apical dendrites of CA1 pyramidal and dentate granule cells at a single point in late gestation, with authors noting a similar stunted appearance of neurite morphology [39]. There are multiple adverse factors that could modify neurite outgrowth, but the potent growth factors BDNF, nerve growth factor and neurotrophin‐3 all actively promote dendritic and synaptic development. Here we noted that BDNF immunoreactivity within the hippocampus of FGR animals was significantly decreased over the course of the third pregnancy trimester, a finding that confirms single timepoint observations in FGR fetal sheep [40] and guinea pigs [39]. Further studies to reveal the growth and other factors, for example inflammatory, that may mediate normal and interrupted dendritogenesis in FGR are warranted so that neuroprotective strategies can be employed.

These results provide novel insights into the cellular‐level neuropathology associated with FGR. Decreased brain volume is a neuropathological feature of FGR, with whole brain, and grey and white matter volumes all reduced in children who experienced FGR [16, 17]. Interestingly, the MRI study of Padilla and colleagues, undertaken at 12 months of age in infants born FGR and appropriate controls, demonstrated a particular vulnerability of grey matter to volume reduction and altered brain organization in infants born FGR [21]. The results of the current study show that, at the cellular level, this is likely underpinned by reduced dendritic arborization during development. We undertook a brain MRI at the mid‐gestation timepoint in this study, 127 days which approximately reflects late‐preterm brain development (32–34 weeks in the human) [22, 28], and found a decrease in whole brain volume in FGR fetal sheep, and a decrease in white matter volume. Overall, while total hippocampal volume was not significantly decreased, results demonstrated a differential volume reduction compared to the thalamus. This result supports other work to indicate regional brain sensitivity of the hippocampus to perinatal insults, including FGR [25, 26, 41]. Indeed, clinical MRI at term‐equivalent age shows reduced hippocampal volume in FGR compared to age‐matched control infants [18], and therefore we might speculate that a later MRI (at the 138 days timepoint) in the current study would detect an overall hippocampal volume reduction. The cellular deficits observed in the current study are likely to persist, given that hippocampal volume remains reduced at 8 years of age in children born FGR [42], with the reduction in hippocampal volume correlated with the degree of growth restriction at birth. The consequences for reduced volume and altered organization of the hippocampus are profound, with this brain region regulating essential cognitive and executive functions [43]. Lodygensky and colleagues quantified this, showing that hippocampal volume loss measured on MRI at term‐equivalent age in FGR infants was correlated with impaired behavioral performance measured at 2 years of age across the domains of attention‐interaction, autonomic and motor functions [18]. Finally, a total reduction in neuronal arborization would also be expected to impact regional circuitry and connectivity. This aligns with the work of Muñoz‐Moreno et al, who showed that brain connectivity deficits were apparent at school age (6 and 10 years of age) in children born FGR, and associated with reduced scores across multiple functional domains, with the most pronounced deficits observed for hyperactivity/inattention and executive function [19]. Together these results provide convincing evidence that the maturational trajectory of neuronal morphology is reduced in the FGR hippocampus, contributing to regional volume deficits that persist after birth, and underpin neurological impairments.

Although we did not observe a significant volume deficit in the hippocampus at the mid‐study timepoint, there was already evidence of reduced CA1 dendrite length, branching and complexity of both apical and basal dendritic arbors. The number of neuronal cells was not affected. We performed ex vivo electrophysiology as a complementary means to quantify functional features of synaptic activity [34]. PPF is widely considered to inversely reflect the probability of neurotransmitter release from presynaptic terminals. The PPF results show that FGR fetuses had less facilitation at short interpulse intervals than control fetuses, a finding that indicates that the FGR offspring had a higher probability of transmitter release. During neurodevelopment, innervating nerves compete for postsynaptic territory. The less successful nerves end up with weaker synapses that have lower probabilities of transmitter release [44, 45]. In the case of FGR lambs, the higher probability of transmitter release could be a consequence of reduced innervating axons having less competition and therefore producing stronger synapses. The input/output data is a measure of the overall effectiveness of presynaptic stimulation and therefore includes assessments of the number of innervating axons as well as the number of synapses and their strengths. At higher stimulus strengths the FGR lambs had smaller maximal responses than controls. Since the PPF data suggests that synapse strength was greater in FGR animals, the combined results likely indicate that reduced FGR outputs at higher stimulus strengths were caused by a reduction in the number of innervating terminals. To further elucidate synaptic potential we performed immunohistochemistry to quantify synapsin‐I in the hippocampus, where synapsin is an integral synaptic vesicle membrane protein that is present in nerve terminals and widely accepted as a measure of total synaptic vesicle pool [46]. Our results demonstrate a significant overall deficit in the FGR group for hippocampal synapsin‐I immunoreactivity, aligning with a total reduction in dendritic spines that are the postsynaptic sites for most excitatory synapses [47]. Combining structural and functional results at the subcellular synaptic level provides insight that a total reduction in dendritic arbors of hippocampal neurons causes a fundamental decrease in cell‐to‐cell connections, with the functional effect of improved efficiency for synapses that are present in the FGR hippocampus.

Apical and basal arbors of CA1 hippocampal neurons were both analyzed in the current study. The longer apical dendritic arbors of hippocampal CA1 neurons project to the *stratum radiatum* and *stratum lacunosum moleculare* layers deep within the hippocampus receiving input via the Schaffer collaterals from CA3 cells, while the shorter basal dendrites project toward the *stratum oriens* providing output to the entorhinal cortex [26]. In the current study, we observed decreased dendrite length, and number of branches, across both apical and basal arbors of the FGR cohort compared to the control group. Sholl analysis for neuronal complexity demonstrated that basal complexity was reduced from 127 days gestation, while apical dendrite complexity was consistently reduced in FGR animals across all gestational timepoints, indicating that apical dendritogenesis may be more highly susceptible to insult. Our results broadly support an earlier study by McClendon et al., who induced a transient hypoxic insult in fetal sheep, resulting in CA1 apical arbors that were more simplified, but this was not the case for basal arbors [48]. Notably, the electrophysiological recordings were undertaken in the CA1 apical region of the Schaffer collaterals, results thus demonstrate concomitant neuronal morphological structure and function disturbances. While apical complexity deficits were more pronounced, the finding that both CA1 apical and basal neurite outgrowth showed an overall reduction in trajectory in FGR animals indicates the cellular basis for hippocampal circuitry to be adversely affected in FGR offspring [49].

This study was conducted in fetal sheep, providing the ability to map normal control and aberrant FGR hippocampal development over the course of the third pregnancy trimester in a long gestation species, and thereby assess the longitudinal effects of placental insufficiency and FGR as would occur in human fetuses. In human pregnancies, estimated fetal weight below the 3rd percentile normalized to growth charts is described as severe FGR [2, 3]. Our model of ovine FGR induces growth restriction that falls within the 5th‐6th percentile for our ovine growth chart [27], thus representing moderate to severe FGR. Blood samples were not collected from fetuses in this study, but we have shown that early‐onset placental compromise induces chronic fetal hypoxia [27]. In contrast to rodents, fetal sheep display a brain maturational profile, grey‐to‐white matter ratio, and cerebral haemodynamics that are similar to humans [50, 51], with peak brain growth occurring prior to birth, albeit white matter maturation peaks earlier in sheep than in humans [28]. In this study, body weight was not different in FGR and control fetuses at 110 days, however, neuropathology was already evident as a decreased apical complexity of the dendritic arbor (Sholl analysis). At the later timepoint of 138 days (0.9 of development) which approximately reflects the term human brain [28], the body weight of the FGR fetuses was 40% less than control fetuses, with brain sparing, and significant arborization deficits. Our study has limitations, including that the nature of the study across gestation and the restricted sample size at each gestation did not permit stratification for fetal sex. We are cognizant of this limitation given that behavioral deficits in children born FGR show sex‐specific effects [52, 53] and preclinical work demonstrates the differential hippocampal structure and functional impairments according to sex [34]. We also acknowledge that in the 127 days group, dendrite analysis and immunohistochemical studies were undertaken in a separate cohort of animals to the brain volume and functional assessments. This was necessitated by different collection techniques for each of the procedures undertaken. Finally, we recognize that both the left and right hemispheres of the hippocampus were used for different outcome measures, where our own hippocampal volume results and previous work have shown differences in susceptibility to insult across the hippocampal hemispheres [25, 26].

## 5 CONCLUSIONS

Neuronal arborization occurs rapidly in the last third of fetal gestation and forms the architectural foundation for hippocampal circuitry and long‐term function in childhood and beyond [26]. This study demonstrates that placental insufficiency causes a severe disruption to dendrite outgrowth in hippocampal neurons of FGR fetuses, which, over time profoundly reduces the trajectory of arborization. Where in the control healthy brain dendrite length was significantly increased by 60% from 0.7 to 0.9 gestation, there was no corresponding significant increase in dendrite length in FGR hippocampal neurons and dendrite arbor complexity was notably reduced, as was the presynaptic protein synapsin. An altered profile of regional growth factor BDNF availability was noted. Total brain volume and white matter volume were reduced in the FGR cohort on MRI at 127 days gestation. The cellular deficits we observed in FGR fetal sheep are very likely to persist after birth or perhaps worsen, given that hippocampal volume is reduced at 8 years of age in children born FGR [42]. Finally, our study confirms functional impairments in synaptic activity of hippocampal neurons within the FGR brain, providing a holistic picture that cellular and regional hippocampal structure and function impairments are all present before birth in FGR infants, laying the foundation for lifelong cognitive and executive function problems, and thus necessitating active investigation of neuroprotective treatments for this population.

## AUTHOR CONTRIBUTIONS

SLM and BJA conceptualized the study. All authors were involved in conducting experiments, performing analysis, and contributed to writing the manuscript.

## CONFLICT OF INTEREST STATEMENT

The authors declare that there are no conflicts of interest.

## Data Availability

Data are available from the corresponding author upon request.
